# A Review of the Latest Evidence on Prognostic Factors in Locally Advanced and Metastatic Urothelial Carcinoma Treated with Immune Checkpoint Inhibitors

**DOI:** 10.3390/medicina62010046

**Published:** 2025-12-26

**Authors:** Ion Cojocaru, Mădălin Guliciuc, Elena Cojocaru, Cristina Serban, Grigore Pascaru, Mihnea Bogdan Borz, Vlad Horia Schitcu, Andrei-Ionut Tise, Iulian Osoianu, Laura-Florentina Rebegea

**Affiliations:** 1 The Clinical Department, The Faculty of Medicine and Pharmacy, “Dunarea de Jos” University of Galati, 800008 Galati, Romania; cojocaruion90@yahoo.com (I.C.); cristina.serban@ugal.ro (C.S.); pascaru.grigore@yahoo.com (G.P.); laura.rebegea@ugal.ro (L.-F.R.); 2 The Department of Urology, “Sfantul Apostol Andrei” Hospital, 800578 Galati, Romania; 3 Reumadiagnostic Clinic, 800402 Galati, Romania; salinschielena@gmail.com; 4 Department of Anatomy, George Emil Palade University of Medicine, Pharmacy, Science, and Technology of Targu Mures, 540142 Târgu Mureș, Romania; borz.m.bogdan@gmail.com; 5 Urology Department, The Oncology Institute “Ion Chiricuţă”, 400015 Cluj-Napoca, Romania; schitcu@yahoo.com (V.H.S.); andrei_tise@yahoo.com (A.-I.T.); 6 Ioan Lascăr Municipal Hospital, 605200 Comănești, Romania; iulianosoianu.20@gmail.com

**Keywords:** urothelial carcinoma, metastatic urothelial carcinoma, immune checkpoint inhibitors, prognostic factors

## Abstract

*Background and Objectives*: Urothelial carcinoma (UC) is one of the most prevalent and lethal cancers worldwide. Identifying and understanding the factors that influence treatment outcome is essential for improving therapeutic effectiveness and predicting patient response. The objective of this review is to estimate how clinical, biochemical, molecular and therapeutic factors impact the prognosis of patients with advanced urothelial carcinoma (aUC) and metastatic urothelial carcinoma (mUC) treated with immune checkpoint inhibitors (ICIs). *Methods*: A review was performed using PubMed, Scopus and Web of Science databases. All articles were published from 2013 to 2025 focusing on prognostic factors in locally advanced and metastatic urothelial carcinoma treated with ICIs. *Results*: Clinical prognostic factors for patients treated with ICIs include poor Eastern Cooperative Oncology Group (ECOG) performance status and the presence of liver or bone metastases, both associated with poor outcomes. Low hemoglobin levels and several biochemical markers, such as high neutrophil-to-lymphocyte ratio (NLR), elevated systemic immune-inflammation index (SII) and low serum sodium are also associated with reduced survival. Programmed cell death-ligand 1 (PD-L1) expression shows predictive relevance for ICI response. Concomitant use of antibiotics or proton pump inhibitors (PPIs) may diminish immunotherapy effectiveness. Additionally, sarcopenia and high lactate dehydrogenase (LDH) levels correlate with poorer clinical outcomes. *Conclusions*: Prognostic outcomes in aUC and mUC are influenced by a complex interaction of clinical, biochemical and molecular factors. Integrative prognostic models are essential to the guidance of personalized immunotherapeutic strategies and the improvement of patient outcomes in aUC and mUC.

## 1. Introduction

Urothelial carcinoma originates from the epithelial cells of the urinary tract. One of the most affected sites is the bladder [1]. Bladder cancer has risen from the 10th to the 9th most frequently diagnosed type of cancer globally. Studies show that both incidence and mortality rates are on the rise. The data indicate that 614,298 people are estimated to have been diagnosed with bladder cancer worldwide in 2022. This percentage shows a 7.1% increase compared with the data reported in 2020 [2]. The highest incidence rates of bladder cancer in both sexes according to GLOBOCAN 2018 were noticed in Southern European countries, as well as in Western Europe and North America [3]. Urothelial bladder cancer is one of the deadliest types of cancers at the global level, with a five-year survival rate of less than 5% in patients who have metastatic disease [4]. Among the factors that lead to a risk of developing bladder cancer is the smoking of tobacco, which is responsible for 50% of cases. Other causes are occupational exposure to ionizing radiation and aromatic amines [5]. For a long time, platinum-based chemotherapy was the standard treatment for chemotherapy-eligible mUC and is still the standard of care in the first-line treatment setting [6]. Later, the therapeutic field for mUC underwent a major transformation with the introduction of ICIs, which have become the standard of care for second-line therapy as well as for first-line treatment in patients who are ineligible for platinum-based chemotherapy [7]. Since 2016, the U.S. Food and Drug Administration (FDA) has authorized two monoclonal antibodies targeting PD-1 (nivolumab and pembrolizumab) and three targeting PD-L1 (atezolizumab, avelumab and durvalumab) for mUC [8].

The appearance of these new drugs has created an urgent need to pinpoint the biomarkers which can identify the patients with highest probability to respond to immunotherapy. In mUC patients treated with ICIs, several important prognostic and predictive markers have been highlighted, including clinical factors, biochemical factors, concomitant medication, molecular factors (PD-L1), sarcopenia, combination between radiotherapy and immunotherapy [7].

The need to identify the potential biomarkers that can help to select the patients who are most likely to benefit from immunotherapy has been clear, as the emergence of these new drugs highlights. In this review, we outline, on one hand, the clinical, molecular, and immune factors that have an influence on prognosis and, on the other hand, the response to immune checkpoint inhibitors in patients with locally advanced and metastatic urothelial carcinoma.

## 2. Materials and Methods

A comprehensive literature search was performed across three major biomedical databases: PubMed, Web of Science and Scopus. The selected studies, published from January 2013 to December 2025, investigated prognostic determinants in patients with locally advanced or metastatic urothelial carcinoma undergoing treatment with immune checkpoint inhibitors. A total of 83 studies were identified, of which 40 were ultimately included based on criteria of relevance and methodological quality. Additionally, some references were included as they offered an essential foundational context about the prognostic factors in patients with mUC who are receiving treatment ICIs. Inclusion criteria were studies which evaluated patients with local aUC and mUC treated with ICIs. Studies involving other cancer types or patients receiving treatments other than ICIs were excluded. A combination of controlled vocabulary terms (MeSH) and keywords related to advanced urothelial carcinoma, metastatic urothelial carcinoma, immune checkpoint inhibitors, prognostic factors were used.

## 3. Prognostic Factors

### 3.1. Clinical Factors

#### 3.1.1. ECOG Performance Status

The ECOG performance status (PS) evaluates the cancer patient’s functional ability and daily living activities using a 0–5 scale, with parameters like self-care, mobility and time spent in bed/chair, indicating overall health and ability to tolerate treatment, with 0 being fully active and 5 being deceased. Ali Raza Khaki et al. demonstrated that there was a greater survival, as far as the overall survival (OS) is concerned, in patients with a PS of 0–1 compared with those with a PS of ≥2 when treated with ICIs in the first line (median 15.2 vs. 7.2 months), but that there was no significant difference in subsequent lines of treatment (median 9.8 vs. 8.2 months). In both treatment lines, there were similarities in terms of the overall response rate (ORR), between PS 0–1 and PS ≥ 2. The ICIs may not counteract the negative prognostic impact of poor PS, especially in the first-line setting, even though the ORR is similar [9]. In a multivariable analysis, ECOG-PS > 0 was identified as a significant independent prognostic factor for both OS and progression-free survival (PFS) [10]. Patients with ECOG-PS > 0 had worse outcomes compared with those with ECOG-PS = 0 [10,11].

#### 3.1.2. Sites of Metastasis

A retrospective study of 917 patients with aUC treated with ICIs evaluated the prognostic significance of metastatic sites in treatment lines. The study demonstrated that the presence of bone and liver metastases in the first line of treatment was associated with shorter PFS, OS and reduced objective response rate (ORR). Similarly, in the second line of treatment or a later setting, bone and liver metastases (LMs) were associated with shorter PFS and OS, and bone-only metastases were additionally associated with lower ORR. Additionally, lymph node-confined metastasis was associated with favorable outcomes in both lines of treatment. Patients with lymph node-only disease had improved PFS, OS and ORR. Overall, these findings demonstrate that metastatic sites are an important prognostic determinant in aUC patients treated with ICIs, with bone and liver involvement predicting poorer outcomes and lymph node-only disease presenting a more favorable prognosis [12]. Another study showed that liver and/or bone metastases was an independent prognostic factor associated with poorer OS and PFS [10,11].

#### 3.1.3. Shorter Time Since Prior Chemotherapy

Shorter time since prior chemotherapy (TFPC) was identified as an independent and significant prognostic factor for both OS and PFS, in patients treated with second-line therapy for aUC. External validation confirmed its strong association with poorer PFS in univariate and most multivariable analyses, and with poorer OS in univariable analyses.

An important conclusion of this study is that a shorter time from prior chemotherapy (<3 months) enhances the possibility to stratify prognosis in patients treated with second-line therapy [10].

#### 3.1.4. Body Mass Index

An ECOG PS ≥ 2, visceral metastases, and a body mass index (BMI) ≥ 25 were strongly associated with overall survival in a multivariate analysis. On the one hand, patients in the high BMI group had significantly better OS compared with those in the low BMI group (21.9 vs. 8.3 months), while median PFS did not show any significant difference between the two groups (4.7 vs. 2.8 months). This study found that a BMI ≥ 25 is an independent predictor of OS in patients with mUC undergoing ICI treatment. On the other hand, it is not a key factor for ORR or PFS. The study also showed that male patients and those without visceral metastases derived greater benefit from ICI treatment in the BMI ≥ 25 group. This is the largest study to investigate the impact of BMI on survival in patients with mUC [13].

#### 3.1.5. Age

Schulz et al. conducted a 99-patient study that included patients who were treated between November 2015 and January 2019. The incidence of immune-mediated adverse events (irAEs) (36.4% vs. 39.4%) and disease control rate (DCR) (59.4% vs. 41.0%) were compared between patients aged ≥ 75 years and those aged < 75 years. Older age was not associated with an increased frequency of irAEs or a lower DCR. The result obtained indicated that ICIs appear to be both safe and effective in the elderly population with mUC, and on the other hand the occurrence of irAEs was associated with a better prognosis [14].

#### 3.1.6. Gender

Another 113-patient study considered, in univariate analysis, aspects such as gender, PFS and OS. Female gender and a worse PFS and OS rate were found [15].

As far as the survival outcome rates are concerned, the studies taken into consideration, such as a systematic review and a meta-analysis that included five studies, showed that females proved to be more likely to have a better ORR than males. Moreover, in the case of the women there was noticed a similar OS to men. As a conclusion to all of these studies, higher response rates and improved survival outcomes in female patients could be noticed. Even so, many studies fail to report gender-specific outcomes [16].

#### 3.1.7. Smoking History

Another approach was to treat patients with mUC with pembrolizumab. This approach was presented in a retrospective multicenter study that utilized data from 95 patients. The primary outcomes showed disease progression and overall mortality. On this occasion, the outcomes were compared based on smoking history and cumulative smoking exposure at the moment of starting of the treatment. No significant relationship was discovered to be between smoking history alone and the effectiveness of pembrolizumab [17].

### 3.2. Biochemical Factors

#### 3.2.1. Hemoglobin and Albumin Level/eGFR Values

Biochemical factors have a complex and indispensable role in understanding, diagnosing, monitoring and treating urological cancers. Their use contributes to increasing survival and personalizing oncological therapies. Looking towards this perspective there were several studies that took into consideration the role of biochemical factors.

For example, an increased OS was demonstrated in a multicenter, retrospective study, in case of high baseline hemoglobin (≥10 g/dL), albumin (≥3.5 g/dL), and eGFR values (≥40 mL/min) [11].

In multivariate analysis, the following aspects were significantly associated with a worse OS and PFS: primary upper tract disease, albumin level < 3.4 g/dL and hemoglobin level < 10 g/dL [15].

Sonpavde et al. identified hemoglobin < 10 g/dL as an important prognostic factor linked with worse OS and PFS in patients treated with second-line therapy for aUC [10].

#### 3.2.2. Hormones Level

Additionally, Lindner et al. demonstrated that elevated luteinizing hormone (LH) and luteinizing hormone/follicle-stimulating hormone (LH/FSH) levels in women, along with high E2 (17β-estradiol) levels in men, could predict improved survival. An increased LH/FSH ratio predicted a better response to ICIs in women. The results obtained by Lindner et al. represent the first clinical evidence suggesting the potential role of sex hormones as prognostic and predictive biomarkers in mUC [18].

#### 3.2.3. Electrolyte Levels

An analysis of two phase 3 trials which included 1787 patients, evaluated the impact of baseline levels of sodium, potassium, chloride, magnesium, and calcium in patients with mUC treated with ICIs. The study demonstrated that serum sodium and chloride levels were linearly associated with clinical outcomes in patients receiving ICIs, whereas potassium, magnesium, and calcium showed no prognostic value. Importantly, elevated baseline sodium (>140 mmol/L) correlated with a higher ORR, exceeding the predictive capacity of tumor PD-L1 expression. These findings indicate that serum sodium level is a simple, inexpensive and widely accessible biomarker with meaningful predictive value for immunotherapy benefit. Incorporating routine electrolyte assessment into clinical decision making may enhance patient selection and optimize treatment strategies in the era of immuno-oncology [19].

#### 3.2.4. CRP Kinetics

A multicenter observational study of 154 patients with mUC treated with ICIs established that early C-reactive protein (CRP) kinetics during ICI therapy strongly predicts treatment response and survival outcomes. Patients presenting a CRP flare response or CRP response had considerably higher ORR and better PFS and OS compared with non-responders, independent of PD-L1 status. Patients who had CRP flare responses had an approximately 70% lower risk of tumor progression and death compared with CRP non-responders. Particularly, prolonged CRP flare responses conferred the greatest clinical benefit. These findings indicate that early CRP dynamics represent a clinically feasible biomarker for optimizing therapy monitoring in mUC [20].

#### 3.2.5. Immune-Inflammatory Biomarkers

The neutrophil–lymphocyte ratio (NLR), recognized as an indicator of stress and systemic inflammation, has received increased interest in recent years because of its prognostic significance for multiple cancers, including urothelial cancer [21].

The prognostic significance of the NLR and the SII was studied based on the Italian SAUL cohort of 267 patients. The researchers analyzed and identified which of these was most effective when combined with PD-L1, with or without LDH. ROC analysis determined the cutoff values for NLR and SII at 3.65 and 884, respectively. Median OS was 14.7 months for NLR < 3.65, compared with 6.0 months for NLR ≥ 3.65; similarly, it was 14.7 months for SII < 884 versus 6.0 months for SII ≥ 884. The combination of SII, PD-L1, and LDH provided better OS stratification than SII plus PD-L1 alone. This led to improved identification of patients with an intermediate prognosis (77% vs. 48%). Significant associations with OS and PFS were confirmed for both the SII + PD-L1 + LDH and SII + PD-L1 combinations in multivariate analyses. The combination of immune-inflammatory biomarkers, including SII, PD-L1, with or without LDH, proved to be a potentially valuable and easy-to-assess prognostic tool that warrants validation to identify patients who could benefit from immunotherapy alone or other treatment options [22].

Shabto et al. demonstrated that elevated values of some indices were associated with poorer clinical outcomes. The indices are NLR, monocyte–lymphocyte ratio (MLR) and platelet–lymphocyte ratio (PLR). In the study by Shabto et al., it was shown that there is a strong correlation between inflammation markers. PLR was found to have the greatest impact on clinical outcomes and on the other hand due to the high correlation between inflammation biomarkers, NLR or MLR could be alternatives to PLR. There is a variant hypothesis regarding the fact that these markers are effective in predicting survival and this is marked by the fact that they may indicate an inability to increase lymphocyte levels as part of the host immune response. The host immune system determined the success of ICIs. Elevated NLR > 5, MLR > 0.55 and PLR > 302 values function as indirect indicators of immune dysregulation in these patients, implying a poor response to ICI treatment. The results of the analysis are in favor of incorporating inflammatory biomarkers into a prognostic model for mUC patients undergoing ICI treatment [23].

Elevated NLR values (>5) were correlated to reduced OS in a separate study [11].

### 3.3. Concomitant Medications

#### 3.3.1. Proton Pump Inhibitors

A meta-analysis highlighted the impact of PPI use on clinical outcomes in patients with urothelial cancer receiving ICI therapy. The analysis showed that PPI use was associated with a 50.7% increased risk of progression and a 58.7% increased risk of death in UC patients receiving ICIs. The result of this meta-analysis indicated that, in patients with urothelial cancer, the concomitant use of PPIs demonstrated significantly lower clinical benefit [24].

Another meta-analysis of ten studies including 3836 patients indicated that concomitant use of PPIs during ICI therapy for aUC and mUC was associated with poorer OS. Subgroup analyses demonstrated similar detrimental effects in patients receiving pembrolizumab and atezolizumab [25].

#### 3.3.2. Antibiotics

A meta-analysis examined the impact of antibiotics (ABs) on the outcomes of patients with solid tumors treated with ICIs, using OS as the primary endpoint and PFS as the secondary endpoint. Across 15 studies, AB exposure before or during ICI therapy was associated with significantly shorter OS. In 13 studies reporting PFS, AB use also correlated with worse outcomes. These findings indicate that antibiotic use significantly reduces both OS and PFS in patients receiving ICIs, suggesting that AB courses should be limited to essential clinical scenarios [26].

One of the most studied aspects has been the impact of antibiotic use in cancer patients receiving ICI treatment. Increasing evidence suggests that the composition of the gut microbiota significantly affects patient prognosis, highlighting a strong interaction between specific immunogenic bacteria and the systemic immune response [27]. From the perspective of the studies reviewed, it appears that the use of antibiotics appears to negatively affect outcomes in patients receiving ICIs by reducing the diversity of the gut microbiota and eliminating key immunogenic bacteria. The same considerations for antibiotics also apply in the case of the concurrent use of proton pump inhibitors. The PPIs commonly used may potentially alter the gut microbiota. Recently, it has been suggested that this interferes with the therapeutic effectiveness of ICIs. Commonly used PPIs can alter the gut microbiota, and they have recently been suggested to interfere with the therapeutic efficacy of ICI [27].

Another meta-analysis of eight studies including 3413 patients evaluated the effect of concomitant AB use on OS in patients with aUC and mUC receiving systemic therapies. Concomitant AB use was associated with significantly worse OS. Subgroup analysis showed that patients receiving atezolizumab with AB experienced poorer OS, whereas no significant difference was observed in those receiving pembrolizumab. In patients treated with chemotherapy alone, AB use did not significantly affect OS [25].

#### 3.3.3. Corticosteroids

A systematic review of five studies comprising 1926 patients evaluated the effect of concomitant steroid therapy OS in patients with aUC and mUC undergoing ICI treatment. Steroid use during ICI therapy was associated with significantly reduced OS compared with non-users. Subgroup analysis showed no statistically significant difference in OS for patients treated with pembrolizumab alongside steroids [25].

Another analysis showed that patients who received cancer-related steroids had a significantly higher risk of disease progression and death. Finally, the study confirmed the association between steroids initially used for cancer-related indications, systemic antibiotics, PPIs, and poorer clinical outcomes with PD-1/PD-L1 checkpoint inhibitors and showed that these treatments may have negative immunomodulatory effects [28].

#### 3.3.4. Opioids

In a cohort of 8870 patients receiving ICIs, early disease progression occurred in 34.2% of patients. Use of opioids was consistently associated with poor survival across all cancer types, while a higher number of concurrent medications correlated with early progression and shorter OS [29].

### 3.4. Molecular Factors

#### Programmed Death Ligand 1 (PD-L1)

The predictive and prognostic value of PD-L1 expression in advanced and metastatic urothelial carcinoma remains inconclusive due to heterogeneity across clinical trials, patient populations, and diagnostic assays. Evidence from first-line trials in cisplatin-ineligible patients suggests that high PD-L1 expression (CPS ≥ 10%) is associated with longer median overall survival with pembrolizumab (KEYNOTE-052) [7,30], whereas low expression may predict poorer outcomes with ICIs (KEYNOTE-361, IMvigor130) [31,32]. Adjuvant and second-line studies further complicate interpretation: IMvigor010 showed no disease-free survival (DFS) benefit with atezolizumab [33], whereas CheckMate 274 and KEYNOTE-045 indicated improved PFS or OS with PD-L1-positive tumors, highlighting the context-dependent nature of PD-L1 as a biomarker [34,35].

In the Danube trial, monotherapy with Durvalumab or the combination of the anti-PD-L1 inhibitor Durvalumab and the anti-CTLA4 agent Tremelimumab proved to offer a superior OS when compared with chemotherapy in patients with high PD-L1 expression. The result shows that the predictive value of high PD-L1 remained unclear [36].

Critically, the variation in PD-L1 assays (22C3, SP142, 28-8) and scoring methods across studies limits cross-trial comparability and may partially explain conflicting results [31,32]. While PD-L1 positivity can guide patient selection, its predictive utility is most pronounced in low-expression subgroups, and it may serve better as a prognostic rather than definitive predictive biomarker. Taken together, PD-L1 expression offers clinically relevant but not definitive guidance, and treatment decisions should integrate tumor biology, patient characteristics, and alternative biomarkers.

### 3.5. Therapy-Related Factors

#### Combined Radiotherapy and Immunotherapy

It is believed that ionizing radiation enhances tumor antigen presentation, triggers cytotoxic T cell activation, and promotes a proinflammatory response. As such, combining immunotherapy with radiotherapy may offer a chance to increase abscopal response rates. The abscopal effect refers to the occurrence of systemic antitumor responses in areas distant from the primary site of irradiation. Even if the mechanisms are unclear, it is believed that it is determined by a systemic immune response against the tumor. Radiotherapy (RT), and its immunomodulatory effects, is being explored as a targeted treatment for the improvement of systemic tumor control. In 2020, 615 clinical trials combining RT and immunotherapy were ongoing, of which 24 focused on patients with muscle-invasive bladder cancer (MIBC). These studies examined the use of ICIs with RT in neoadjuvant or concomitant settings, as well as maintenance therapy after trimodal therapy (TMT), potentially improving outcomes and providing more options for patients, especially those too frail to undergo chemotherapy or surgery [37].

A retrospective cohort study realized by Fukushima et al. analyzed the results of pembrolizumab monotherapy combined with radiotherapy in the case of 14 pre-treated mUC patients. It was demonstrated that the radiotherapy group had a significantly higher ORR (65% vs. 19%) and a higher one-year PFS rate (52% vs. 28%) compared with patients who did not receive irradiation [38].

### 3.6. Host Factors

#### Sarcopenia

Sarcopenia refers to the loss of skeletal muscle mass and function, and it is common in cancer patients, especially patients with metastatic or advanced stages. Sarcopenia has been linked to poorer outcomes in chemotherapy, surgery, or immunotherapy patients; to heightened chemotherapy toxicity; and to reduced quality of life, although the adverse effects of ICIs are not consistent proof for this [39]. In order to have a clear view, the skeletal muscle index was calculated by dividing the total muscle volume at the L3 vertebra level by the square of the height. To perform sarcopenia diagnosis, gender-specific thresholds were used, with values below 45.4 cm^2^/m^2^ for males and 34.4 cm^2^/m^2^ for females. What could be noticed was that patients with sarcopenia had significantly lower PFS compared with those without it (2.7 vs. 6.7 months). Univariable Cox regression analysis identified several significant prognostic factors for poor PFS, including female gender, sarcopenia, ECOG PS ≥ 2, and elevated LDH levels [40]. So far, studies have not indicated that sarcopenia is an obstacle to immunotherapy, but further clinical trials are needed to confirm these findings due to limited case data [39].

The prognostic factors in aUC and mUC treated with ICIs are summarized in Table 1.

## 4. Conclusions

Bladder cancer represents a major oncological burden worldwide, characterized by a steadily increasing incidence and mortality despite notable advances in systemic therapy. The prognosis of patients with advanced or metastatic urothelial carcinoma treated with immune checkpoint inhibitors is determined by a combination of clinical, biological, therapeutic and host-related variables. Among these, performance status, metastatic sites, and baseline biochemical markers remain the most reliable predictors of outcome. Poor ECOG status, liver or bone metastases, anemia, hypoalbuminemia, and elevated systemic inflammation consistently correlate with reduced survival.

Several concomitant medications—including proton pump inhibitors, antibiotics, corticosteroids, and opioids—are associated with diminished immunotherapy efficacy, likely through immunological and microbiome-mediated mechanisms. Their use should therefore be carefully justified during ICI treatment.

PD-L1 expression, although extensively studied, offers inconsistent predictive value due to assay variability and heterogeneous trial results. While it may support therapeutic decisions in select settings, it cannot yet function as a definitive standalone biomarker.

Emerging markers such as CRP kinetics, serum sodium and immune-inflammatory biomarkers show promise for improving prognostic assessment and are easily applicable in clinical practice. Host-related factors like sarcopenia also contribute to outcome variability but do not appear to compromise the effectiveness of ICIs.

Taken together, current evidence indicates that the optimal management of urothelial carcinoma requires an integrated approach combining clinical, biochemical, and molecular data. Future efforts should focus on developing validated, multifactorial prognostic models to refine patient selection and enhance the therapeutic benefit of immunotherapy.

## Figures and Tables

**Table 1:** Prognostic factors and their predictive value in aUC and mUC patients, with a particular focus on ICIs.

Category	Factor	Impact on Outcome	Key Observations/Notes
Clinical Factors	ECOG Performance Status	Poor PS (≥2) → worse OS; ORR similar	Prognostic, especially in first-line ICI therapy [9,10,11]
	Sites of Metastasis	Liver/bone → shorter PFS & OS, lower ORR; LN-only → longer PFS & OS, higher ORR	Both first- and later-line treatment relevant [10,11,12] Independent of ECOG, Hb, liver metastases [10]
	Time since prior chemotherapy (TFPC)	Shorter TFPC → poorer OS & PFS	
	Body Mass Index (BMI)	BMI ≥ 25 → better OS; no effect on PFS/ORR [13]	
	Age	≥75 years → similar irAEs and DCR	ICIs safe and effective in elderly [14]
	Gender	Female → higher ORR; OS similar	Many studies lack gender-specific analysis [15,16]
	Smoking History	No significant effect [17]	
Biochemical Factors	Hemoglobin level	Hb < 10 g/dL → poorer OS	Independent prognostic marker [10,11,15]
	Albumin level	Albumin < 3.5 g/dL → poorer OS [10,11,15]	
	eGFR	eGFR < 40 mL/min → poorer OS [10,11,15]	Supports renal function monitoring
	Sex Hormones	High LH/FSH ratio in women, high E2 in men → improved survival & response	First clinical evidence of predictive role [18]
	Electrolytes (Na, Cl, K, Mg, Ca)	High sodium (>140 mmol/L) → higher ORR, improved OS & PFS; chloride also correlates; K, Mg, Ca → no correlation	Sodium stronger predictor than PD-L1 [19]
	CRP kinetics	CRP flare/responder → higher ORR, improved PFS & OS	Independent of PD-L1 status; early monitoring valuable [20]
	NLR;MLR;PLR; SII	NLR < 3.65, SII < 884 → longer OS; elevated NLR, MLR, PLR → poor outcomes	SII + PD-L1 ± LDH improves risk stratification [11,22,23]
Concomitant Medications	Proton Pump Inhibitors	PPI use → ↑ risk of progression and death, ↓ ORR	Interferes with gut microbiota, reduces ICI efficacy [24,25]
	Antibiotics	AB use → shorter OS & PFS	Timing of use critical; impacts gut microbiome [25,26,27]
	Corticosteroids	Significantly reduced OS, higher risk of disease progression and death	May have negative immunomodulatory effects [25,28]
	Opioids	Poor survival [29]	
Molecular Factors	PD-L1 Expression	High PD-L1 expression → improved OS/response in some trials; low PD-L1 expression → worse outcome	Predictive role inconsistent; assay-dependent [7,30,31,32,33,34,35,36]
Therapy Related Factors	Radiotherapy + Immunotherapy	RT + ICI → higher ORR and 1-year PFS rate	Abscopal effect may enhance systemic response [37,38]
Host Factors	Sarcopenia	Presence → lower PFS	Not yet shown to limit ICI efficacy; needs further study [39,40]

The symbol “↑” indicates an increase. The symbol “↓” indicates an decrease.

## Data Availability

No additional data were generated given that this is a literature review.

## Abbreviations

The following abbreviations are used in this manuscript:

mUC	Metastatic urothelial carcinoma
aUC	Advanced urothelial carcinoma
ICIs	Immune checkpoint inhibitors
PPIs	Proton pump inhibitors
PD-1	Programmed cell death protein 1
PD-L1	Programmed cell death-ligand 1
ECOG	Eastern Cooperative Oncology Group
NLR	Neutrophil-to-lymphocyte ratio
SII	Systemic immune-inflammation index
LDH	Lactate dehydrogenase
FDA	U.S. Food and Drug Administration
PS	Performance status
OS	Overall survival
ORR	Overall response rate
UC	Urothelial carcinoma
PFS	Progression free survival
LM	Metastases
Hb	Hemoglobin
TFPC	Time from prior chemotherapy
BMI	Body mass index
irAEs	Immune-mediated adverse events
DCR	Disease control rate
LN	lymph node
LH	luteinizing hormone
FSH	follicle-stimulating hormone
MLR	monocyte–lymphocyte ratio
PLR	platelet–lymphocyte ratio
CRP	C-reactive protein
MeSH	Medical subject headings
RT	Radiotherapy
MIBC	Muscle-invasive bladder cancer
AB	Antibiotics
DFS	Disease-free survival
